# Characterization and comparative analysis of transcriptional profiles of porcine colostrum and mature milk at different parities

**DOI:** 10.1186/s12863-021-00980-5

**Published:** 2021-08-10

**Authors:** Brittney N. Keel, Amanda K. Lindholm-Perry, William T. Oliver, James E. Wells, Shuna A. Jones, Lea A. Rempel

**Affiliations:** USDA-ARS Roman L Hruska US Meat Animal Research Center, Clay Center, NE 68933 USA

**Keywords:** RNA-Seq, Transcriptome, Milk, Colostrum, Total RNA, Gene expression, Long non-coding RNA, Lancaster method

## Abstract

**Background:**

Porcine milk is a complex fluid, containing a myriad of immunological, biochemical, and cellular components, made to satisfy the nutritional requirements of the neonate. Whole milk contains many different cell types, including mammary epithelial cells, neutrophils, macrophages, and lymphocytes, as well nanoparticles, such as milk exosomes. To-date, only a limited number of livestock transcriptomic studies have reported sequencing of milk. Moreover, those studies focused only on sequencing somatic cells as a proxy for the mammary gland with the goal of investigating differences in the lactation process. Recent studies have indicated that RNA originating from multiple cell types present in milk can withstand harsh environments, such as the digestive system, and transmit regulatory molecules from maternal to neonate. Transcriptomic profiling of porcine whole milk, which is reflective of the combined cell populations, could help elucidate these mechanisms. To this end, total RNA from colostrum and mature milk samples were sequenced from 65 sows at differing parities. A stringent bioinformatic pipeline was used to identify and characterize 70,841 transcripts.

**Results:**

The 70,841 identified transcripts included 42,733 previously annotated transcripts and 28,108 novel transcripts. Differential gene expression analysis was conducted using a generalized linear model coupled with the Lancaster method for *P*-value aggregation across transcripts. In total, 1667 differentially expressed genes (DEG) were identified for the milk type main effect, and 33 DEG were identified for the milk type x parity interaction. Several gene ontology (GO) terms related to immune response were significant for the milk type main effect, supporting the well-known fact that immunoglobulins and immune cells are transferred to the neonate via colostrum.

**Conclusions:**

This is the first study to perform global transcriptome analysis from whole milk samples in sows from different parities. Our results provide important information and insight into synthesis of milk proteins and innate immunity and potential targets for future improvement of swine lactation and piglet development.

**Supplementary Information:**

The online version contains supplementary material available at 10.1186/s12863-021-00980-5.

## Background

Colostrum and milk play a key role in survival and growth of the neonate, providing essential nutrients and antibodies [1]. Langer et al. [2] investigated differences in composition of colostrum and mature milk in several eutherian species and found that in some species colostrum contains higher concentrations of proteins than mature milk, and in other species the fluids have similar composition. These differences are likely due to species-specific strategies for immunoglobulin transfer, i.e. prenatal transfer via placenta or yolk sac versus postnatal transfer via colostrum [2]. The critical importance of colostrum and milk for the newborn piglet has been well-documented [1, 3].

Piglet growth and survival are critical to the swine industry. Progeny born to primiparous sows (gilts) are born lighter, grow slower, and have higher mortality rates than those born to multiparous sows [4, 5]. It has been hypothesized that differences in lifetime performance between gilt progeny and sow progeny may be due to differences in lactation performance, specifically lower levels of immunoglobulin G (IgG) and other energetic components in the colostrum and milk of gilts. However, data from Craig et al. [6] showed no parity differences in total IgG, fat, protein, lactose, and net energy concentrations. These results suggest that the poorer performance of gilt progeny is unlikely due to insufficient nutrient levels and is more likely due to differences in colostrum and milk intake and their ability to digest and absorb each component [5].

The presence of many different ribonucleic acid (RNA) types, including messenger RNA (mRNA), micro RNA (miRNA), long non-coding RNA (lncRNA), and circular RNA (circRNA) has been documented in milk from several mammalian species [7–12]. In fact, the total RNA concentration in human breast milk was higher than in other body fluids [8]. Whole milk contains many different cell types, including mammary epithelial cells (MEC), neutrophils, macrophages, and lymphocytes [7, 13], as well nanoparticles, such as milk exosomes [14]. Products from exosomes can withstand harsh environments such as the digestive system and allow for transmission of regulatory molecules (e.g., miRNA) from maternal to neonate [15–17]. Additionally, mRNA that are resistant to acidic conditions and RNase treatments have been identified in bovine milk [15, 18].

A limited number of livestock transcriptomic studies have reported sequencing of milk, including two in swine [19, 20], three in cattle [21–23], one in goat [24], one in sheep [25], and one in buffalo [26]. The emphasis of these studies was gene expression related to the lactation process, and as such, milk somatic cells were sequenced as a proxy for the mammary gland tissue. Additionally, the RNA repertoire derived from milk exosomes has been reported in cattle [11, 27] and swine [12, 28]. To our knowledge, there have been no studies that have reported direct sequencing of porcine whole milk samples.

As the only nutritional source for newborn piglets, porcine colostrum and milk contain critical nutritional and immunological components, including carbohydrates, lipids, and immunoglobulins, as well as exosomes, oligosaccharides, and bacteria, which possibly act as biological signals and modulate the intestinal environment and immune status later in life [29]. As part of an effort to explore the transcriptomic profile of the piglet’s neonatal diet, we performed total RNA-sequencing (total RNA-Seq) on porcine whole milk samples (colostrum and mature milk) from dams in parities one through four to characterize and compare the two transcriptomes. We identified novel mRNA and lncRNA transcripts and quantified expression of both known and novel porcine transcripts. Expression profiles were compared to identify differentially expressed genes (DEG) between colostrum and mature milk between parities.

## Results

### High-throughput sequencing

**Fig. 1:**
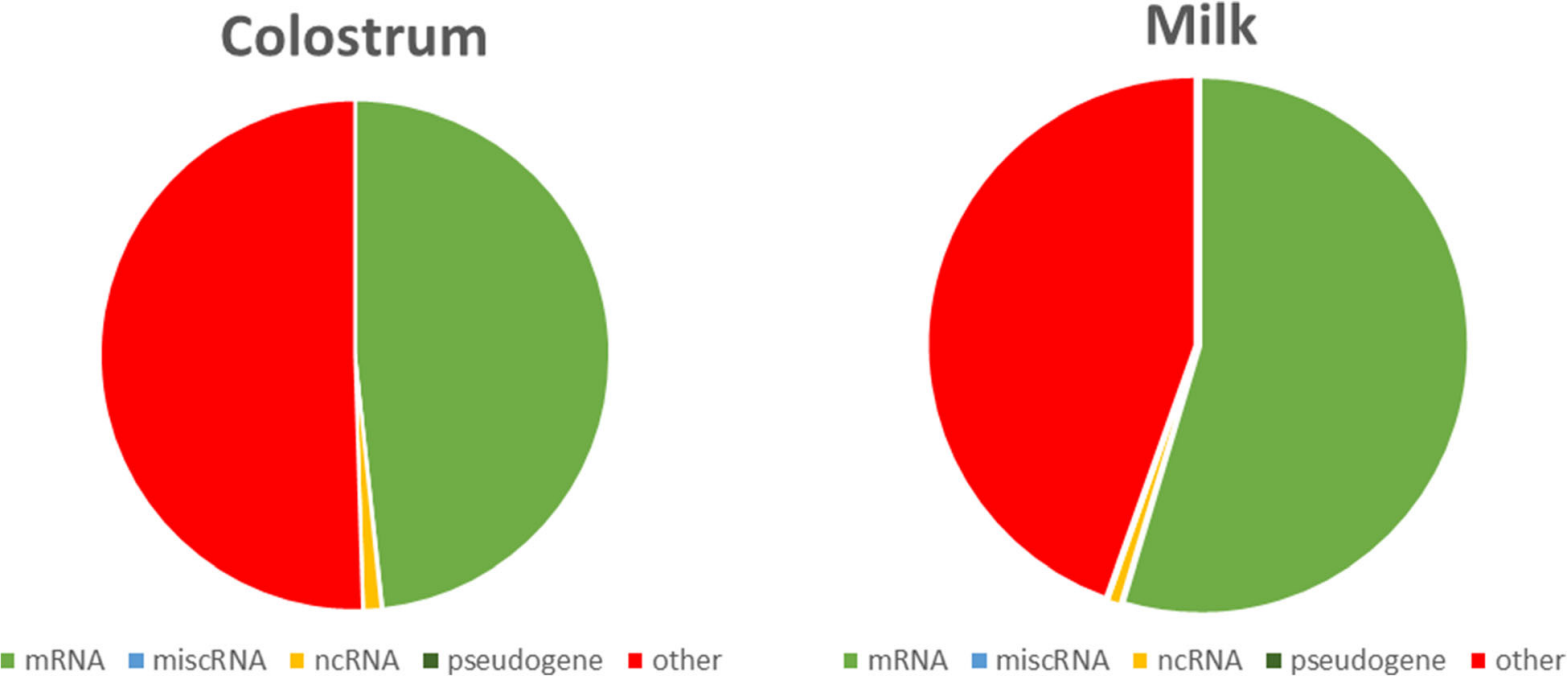
Distribution of reads aligning to the *S. scrofa* 11.1 genome. RNA classifications are based on the *S. scrofa* reference genome annotation (NCBI Release 106)

RNA-Seq libraries were sequenced generating over 6 billion 75 base pair (bp) paired-end reads, with an average of 46.2 million reads per library (Table S1). The number of reads in the colostrum libraries ranged from 22.6 to 81.8 million reads with an average of 44.4 million reads, while the number of reads in the mature milk libraries ranged from 24.2 to 97.8 million reads with an average of 48.0 million reads. After adapter removal and read trimming, the resulting high-quality reads were mapped to the Sscrofa 11.1 genome assembly with an average 99.6% read mapping rate per library. The number of reads aligning to known mRNA, miscellaneous RNA (miscRNA; short non-coding RNA), non-coding RNA (ncRNA), and pseudogenes in the swine genome are presented in Table S2. It was observed that ~ 50% of reads mapped to known mRNA, while 50.5% of colostrum reads and 44.5% of milk reads were mapped outside of annotated loci, potentially harboring novel transcripts (Fig. 1).

### Transcript identification and characterization

**Fig. 2:**
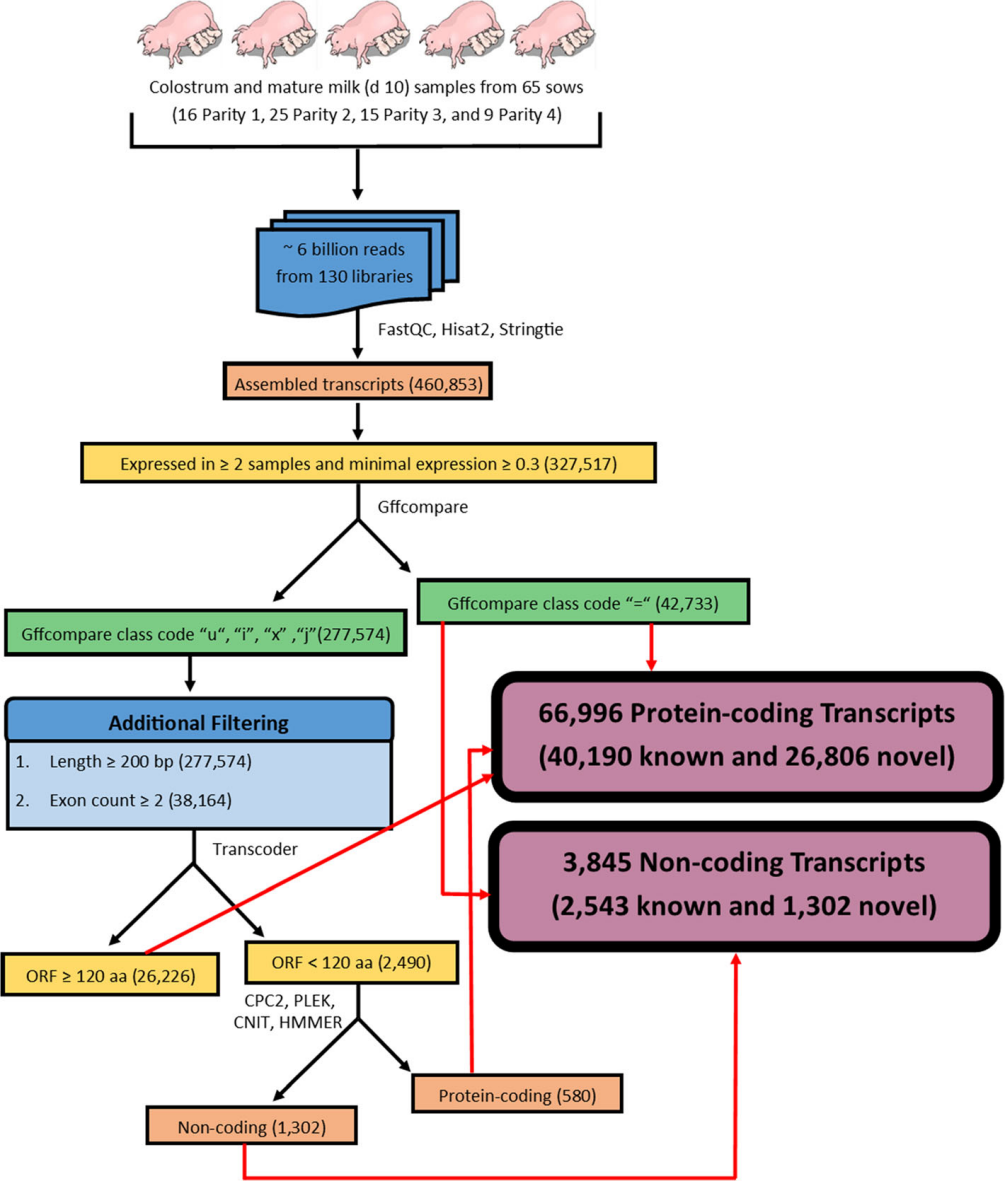
Computational pipeline used to determine novel transcripts from RNA-Seq data

Transcripts, assembled individually for each library, were merged into a single set of 460,853 putative transcripts. This set was subjected to several filtering steps to remove transcriptional noise and classify transcripts (Fig. 2). Transcripts identified in only one library and lowly expressed transcripts were removed, as these were considered transcriptional noise. The remaining set of transcripts was filtered to include only those with class codes ‘=’, ‘u’, ‘x’, ‘j’, and ‘i’ (Figure S1). The transcripts with class codes ‘u’, ‘x’, ‘j’, and ‘i’ were further filtered by length, and number of exons. This set of 38,164 putative novel transcripts were then subjected to classification by open reading frame (ORF) length and protein coding potential score to complete transcript characterization. In total, 70,841 transcripts were identified in the porcine milk transcriptome, including 42,733 previously annotated transcripts as well as 28,108 novel transcripts.

**Fig. 3:**
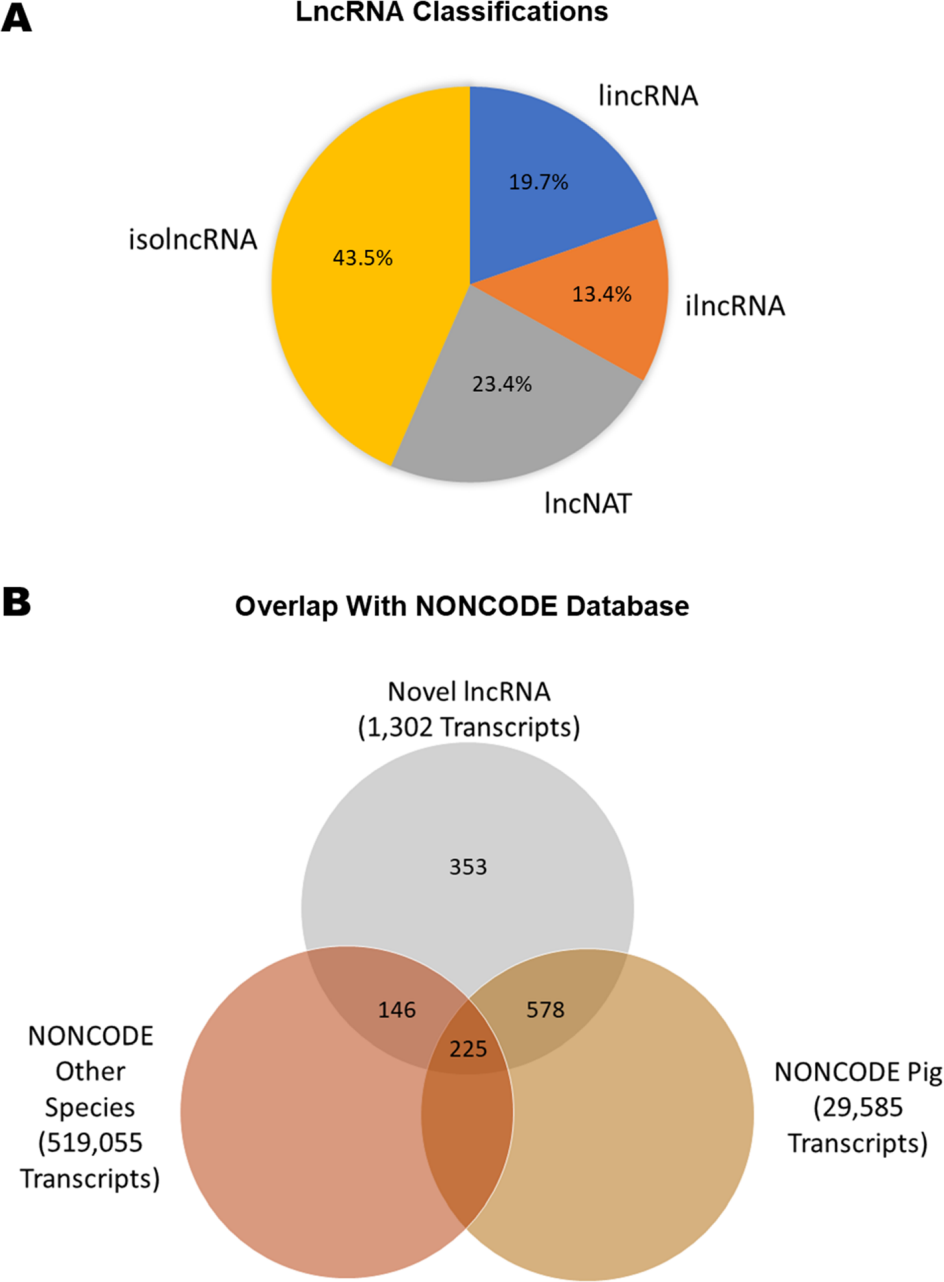
Classification of novel lncRNA In (**A**) lincRNA denotes intergenic long-noncoding RNA, ilncRNA denotes intronic long-noncoding RNA, lncNAT denotes long non-coding antisense transcripts, and isolncRNA denotes novel isoform long non-coding RNA

**Fig. 4:**
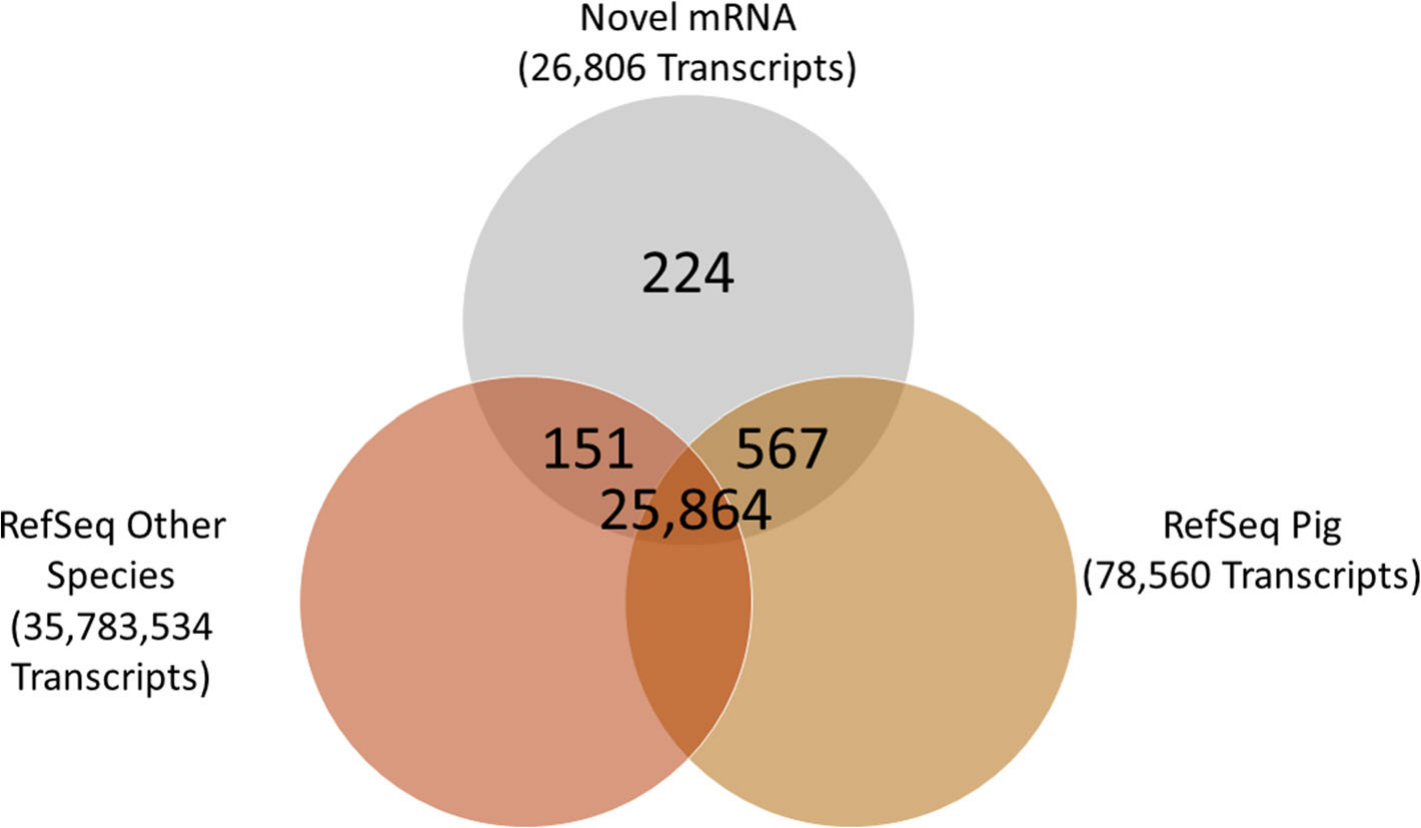
Overlap of novel protein-coding transcripts with RefSeq database

Genomic coordinates of the identified novel transcripts are given in Tables S3 and S4. Among the novel lncRNA transcripts, 256 and 175 were intergenic long non-coding RNA (lincRNA) and intronic long non-coding RNA (ilncRNA), respectively, while 305 lncRNA flanked a protein-coding gene in a divergent orientation (long non-coding natural antisense transcripts; lncNAT) and 566 were novel isoform long non-coding RNA (isolncRNA) (Fig. 3A). Using the BLAST algorithm, a total of 578 lncRNA exhibited homology with transcripts in the porcine NONCODE database, 146 lncRNA exhibited homology with non-coding transcripts in other species, and 225 lncRNA were homologous to noncoding transcripts in both swine and other species (Fig. 3B; Table S5). A similar analysis identified that 26,582 of the novel mRNA transcripts were homologous to known transcripts in swine and other species (Fig. 4).

**Fig. 5:**
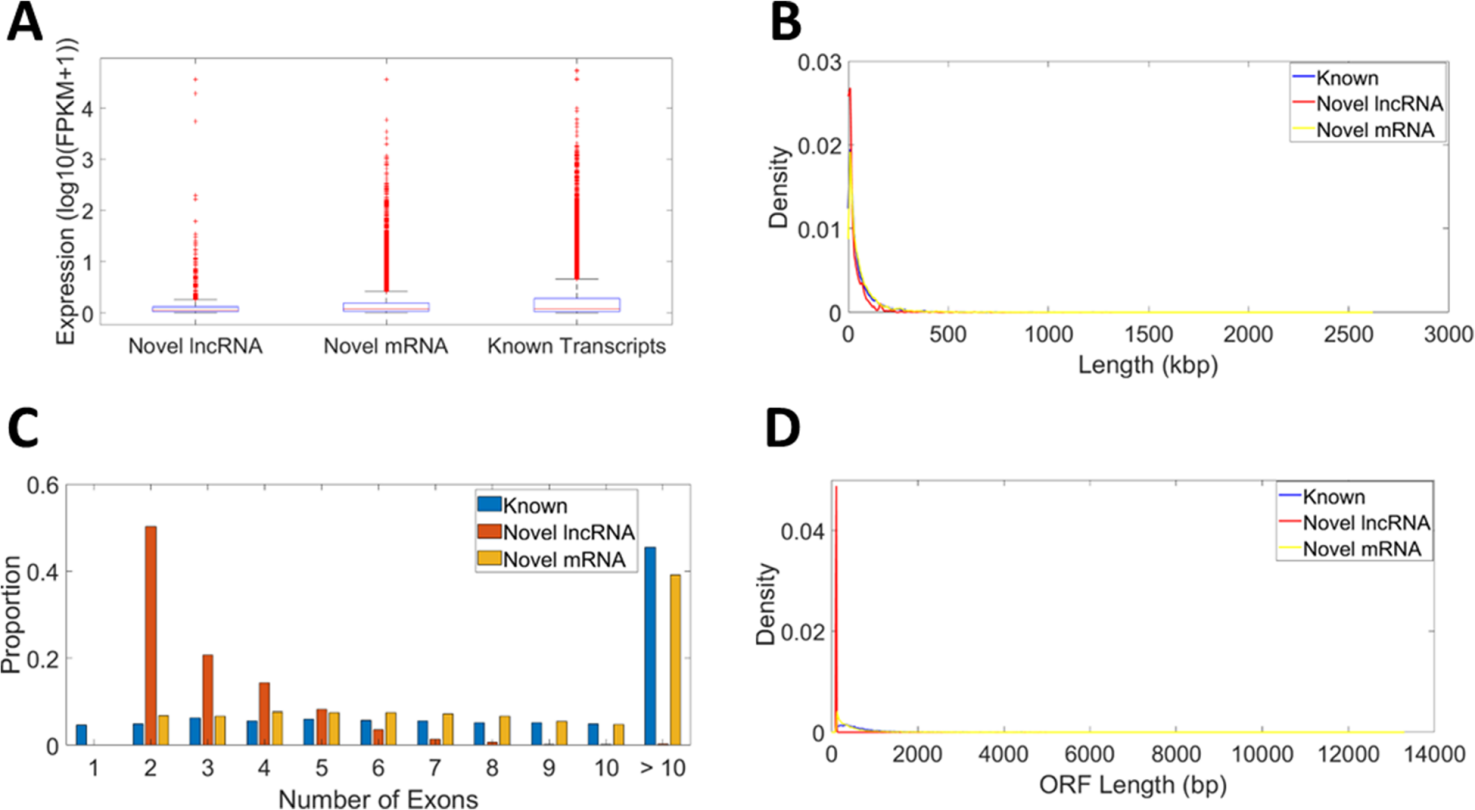
Basic features of transcripts. **A** Expression level of transcripts. **B** Length distribution of transcripts. **C** Number of exons for transcripts. **D** ORF length distribution of transcripts

Basic sequence features of the novel transcripts, including length, exon number, expression, and ORF length, are shown in Fig. 5 and Table 1. Novel lncRNA were significantly shorter and expressed at lower levels than novel mRNA and known transcripts (Fig. 5A, B). The exon number of the novel lncRNA and coding transcripts were notably smaller than that of known transcripts (Fig. 5C). The ORF length of novel lncRNA was significantly shorter than ORF length in known and novel coding transcripts, while the ORF length of novel coding transcripts was significantly shorter than that of known transcripts (Fig. 5D).

**Table 1:** Median characteristics of expressed transcripts

	Novel lncRNA	Novel Coding	Known Transcripts
**Expression**^a^	0.06^d, f^	0.09^e^	0.09
**Length**^b^	13.37^e, f^	34.51	29.25
**Number Exons**	2^e, f^	8^e^	10
**ORF Length**^c^	109^e, f^	332^e^	481

^a^ Measured in log_10_(FPKM+ 1)

^b^ Measured in kbp

^c^ Measured in bp

^e^ Left-tailed Wilcoxon rank-sum *P*-value < 0.05 compared to known transcripts

^f^ Left-tailed Wilcoxon rank-sum *P*-value < 0.05 compared to novel coding

**Fig. 6:**
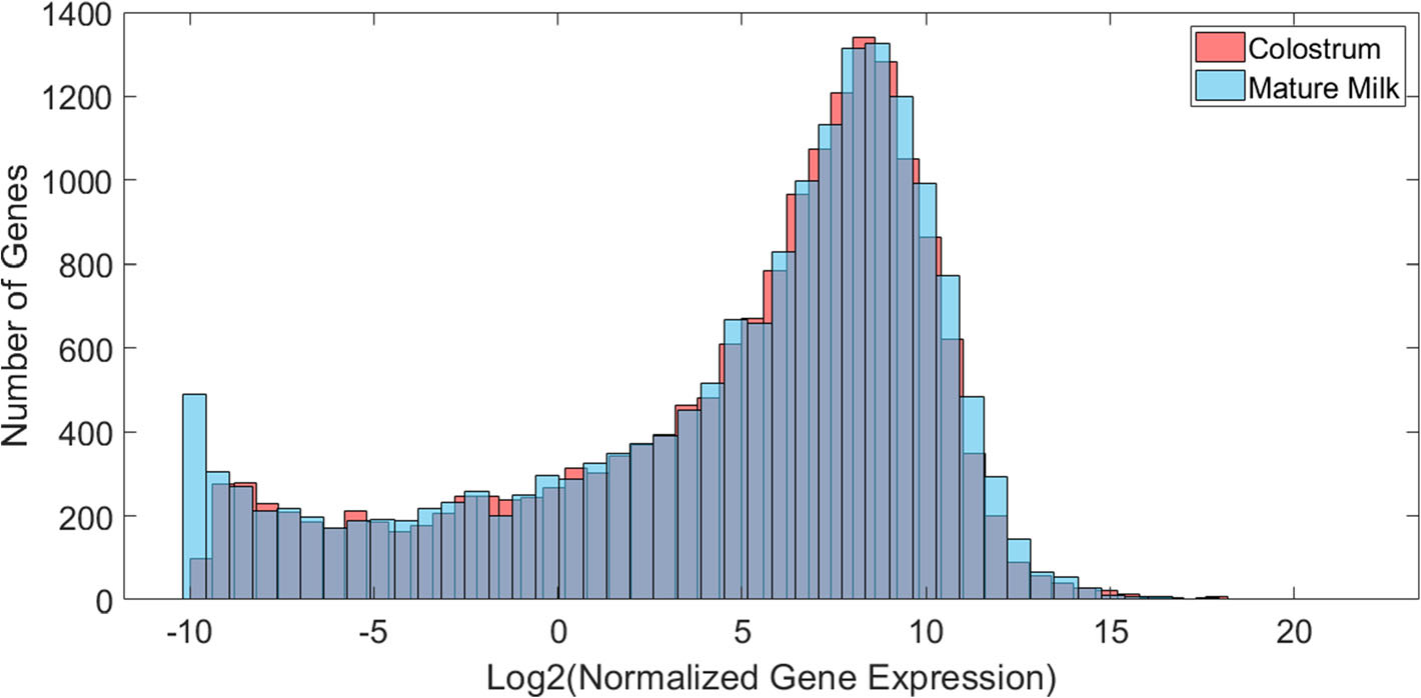
Plot of gene expression distribution for colostrum and mature milk samples. Values are averaged across samples in each group

**Fig. 7:**
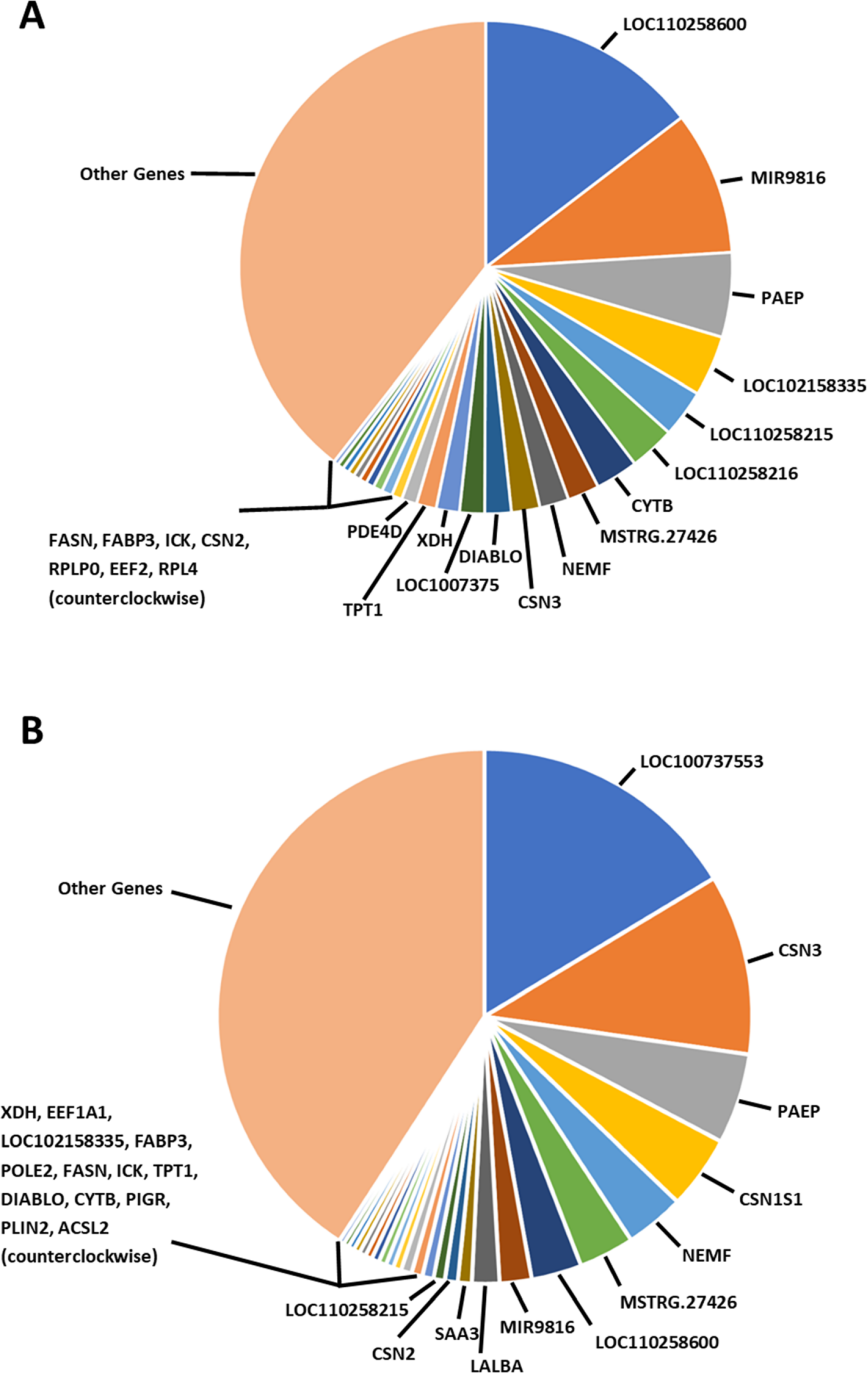
Relative gene abundances of highest expressed genes in **A** colostrum and **B** mature milk samples

Transcripts corresponded to 17,910 unique gene loci, of which 17,296 genes were previously annotated in the *S. scrofa* reference genome. Previously annotated transcripts corresponded to 16,992 known gene loci, while unannotated protein-coding and non-coding transcripts corresponded to 8384 (7933 known) and 1059 (843 known) loci, respectively. In general, gene expression values were widely distributed (Fig. 6), with the distributions of gene expression values being approximately equal for colostrum and mature milk. There was a large overlap (19 out of 25) in the top twenty-five most abundantly expressed genes in colostrum and mature milk (Table 2; Fig. 7).

**Table 2:** Top expressed genes in porcine colostrum and mature milk

Gene Symbol	Description	Colostrum^a^	Mature Milk^a^
LOC110258600	Basic salivary proline-rich protein 2-like	28.49 (1)	8.33 (7)
MIR9816	Uncharacterized ncRNA	18.39 (2)	5.30 (8)
PAEP	Progestagen associated endometrial protein	10.85 (3)	15.01 (3)
LOC102158335	Uncharacterized ncRNA	7.77 (4)	1.77 (15)
LOC110258215	Progesterone receptor-like	6.10 (5)	1.87 (12)
LOC110258214	Basic salivary proline-rich protein 4-like	5.83 (6)	0.712 (29)
CYTB	Cytochrome b	5.46 (7)	1.05 (22)
MSTRG.27426	Novel mRNA	4.06 (8)	9.49 (6)
NEMF	Nuclear export mediator factor	3.74 (9)	9.81 (5)
CSN3	Casein kappa	3.65 (10)	29.90 (2)
DIABLO	Diablo IAP-binding mitochondrial protein	3.38 (11)	1.10 (21)
LOC100737553	Peptidyl-prolyl cis-trans isomerase A pseudogene	3.20 (12)	44.80 (1)
EEF1A1	Eukaryotic translation elongation factor 1 alpha 1	2.98 (13)	1.83 (14)
CSN1S1	Casein alpha s1	2.54 (14)	12.12 (4)
LOC102163473	Uncharacterized ncRNA	1.96 (15)	0.58 (??)
XDH	Xanthine dehydrogenase	1.35 (16)	1.86 (13)
TPT1	Tumor protein, translationally-controlled 1	1.34 (17)	1.12 (20)
PDE4D	Phosphodiesterase 4D	1.19 (18)	0.25 (49)
FASN	Fatty acid synthase	1.05 (19)	1.27 (18)
FABP3	Fatty acid binding protein 3	0.94 (20)	1.35 (16)
ICK	Ciliogenesis associated kinase 1	0.87 (21)	1.25 (19)
CSN2	Casein beta	0.82 (22)	2.13 (11)
RPLP0	Ribosomal protein lateral stalk subunit P0	0.82 (23)	0.36 (41)
EEF2	Eukaryotic translation elongation factor 2	0.78 (24)	0.42 (38)
RPL4	Ribosomal protein L4	0.63 (25)	0.30 (42)
LALBA	Lactalbumin alpha	0.36 (49)	4.54 (9)
SAA3	Serum amyloid A-3 protein	0.25 (76)	2.30 (10)
POLE2	DNA polymerase epsilon 2, accessory subunit	0.57 (27)	1.35 (17)
PIGR	Polymeric immunoglobulin receptor	0.11 (148)	1.00 (23)
PLIN2	Perilipin 2	0.06 (257)	0.89 (24)
ACSL3	Acyl-CoA synthetase long chain family member 3	0.13 (132)	0.87 (25)

^a^Average normalized gene expression value (× 10^5^) across samples. Number in parenthesis is ranking in expressed genes

### Expression of cell-specific markers

**Fig. 8:**
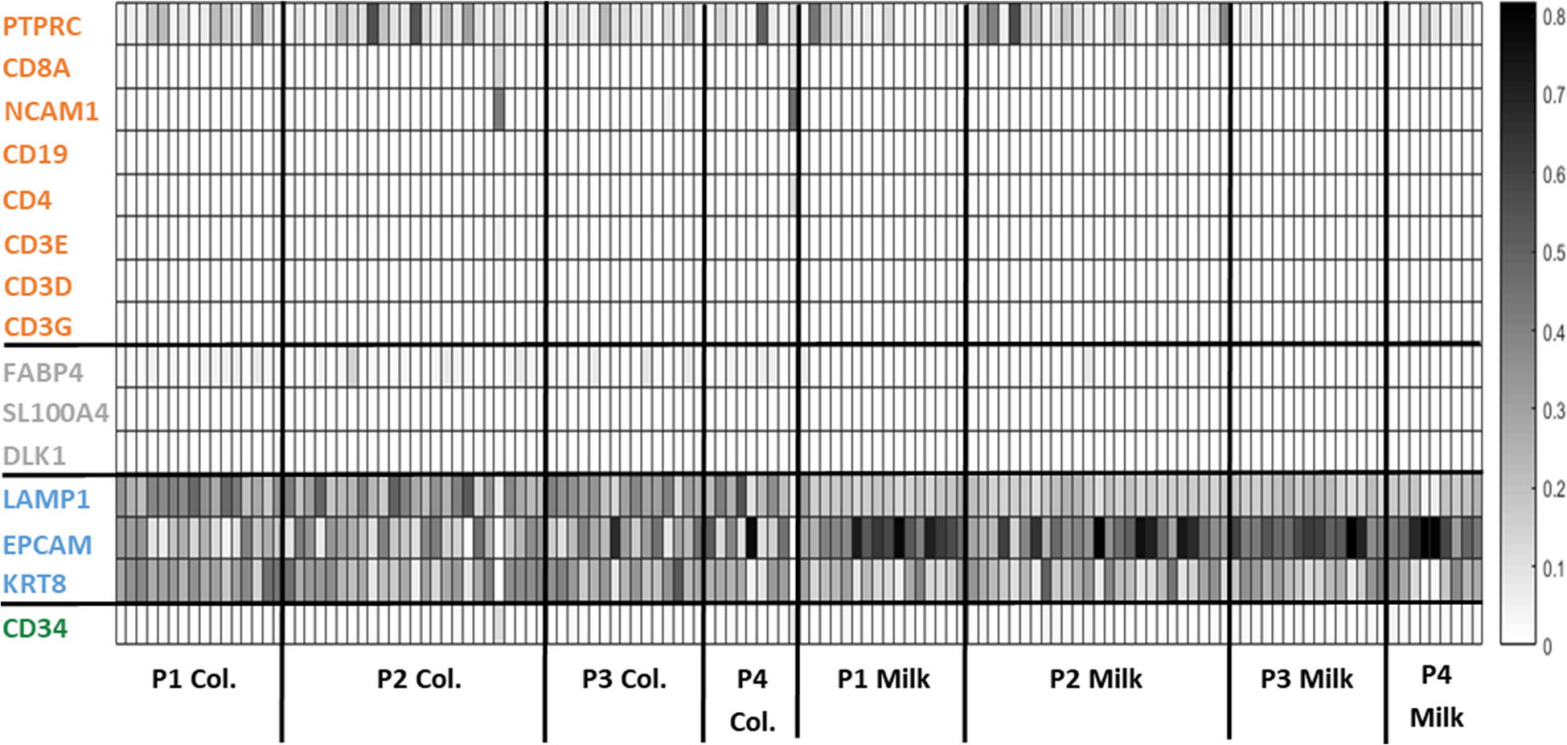
Expression of cell-specific markers in colostrum and mature milk transcriptomes. Each box in the heatmap represents the relative proportion of cell-specific marker in the sample, i.e. the number of reads mapped to the cell-specific marker divided by the sum of the reads mapped to cell-specific markers. Samples are organized by milk type (colostrum and milk) and parity (P1-P4) as shown on the x-axis. Cell-specific markers are shown along the y-axis, with font color indicating the cell marker type: Green = stem cell, Blue = epithelial cell, Gray = stromal cell, and Orange = immune cell

Whole milk is a complex fluid containing a heterogenous mixture of cells [30, 31]. Analysis of gene expression of cell-specific markers, the same markers utilized in [32], was used to estimate the proportion of various cell types present in colostrum and mature milk samples (Table 3; Fig. 8). Epithelial cells were the most abundant cells in all samples, with higher abundance in mature milk samples. Stromal cells represented ~ 1% of the cell population in all samples. Immune cells and stromal cells were both more abundant in colostrum samples.

**Table 3:** Average proportion of cell types in colostrum and mature milk samples

Cell Type^a^	P1 Col.	P2 Col.	P3 Col.	P4 Col.	P1 Milk	P2 Milk	P3 Milk	P4 Milk
Pan- Immune (PTPRC)	0.100	0.136	0.088	0.112	0.087	0.134	0.037	0.056
Immune (CD8A)	0.001	0.007	0.002	0.011	0.001	0.000	0.001	0.000
Immune (NCAM1)	0.002	0.018	0.003	0.052	0.000	0.000	0.000	0.000
Immune (CD19)	0.000	0.000	0.000	0.000	0.000	0.000	0.000	0.000
Immune (CD4)	0.001	0.001	0.000	0.012	0.000	0.000	0.000	0.000
Immune (CD3E)	0.001	0.003	0.000	0.004	0.000	0.000	0.000	0.000
Immune (CD3D)	0.002	0.004	0.003	0.006	0.002	0.002	0.002	0.001
Immune (CD3G)	0.000	0.000	0.000	0.001	0.000	0.000	0.001	0.000
Stromal (FABP4)	0.029	0.025	0.023	0.026	0.006	0.008	0.003	0.003
Stromal (SL100A4)	0.003	0.002	0.002	0.002	0.000	0.000	0.000	0.000
Stromal (DLK1)	0.000	0.001	0.000	0.003	0.000	0.000	0.000	0.000
Epithelial (LAMP1)	0.333	0.296	0.295	0.261	0.177	0.168	0.172	0.160
Epithelial (EPCAM)	0.020	0.236	0.291	0.294	0.516	0.449	0.534	0.558
Epithelial (KRT8)	0.311	0.259	0.284	0.202	0.198	0.223	0.234	0.203
Stem (CD34)	0.010	0.012	0.009	0.015	0.010	0.013	0.014	0.016

^a^ Cell-specific marker shown in parentheses

### PCA and differential expression analysis

**Fig. 9:**
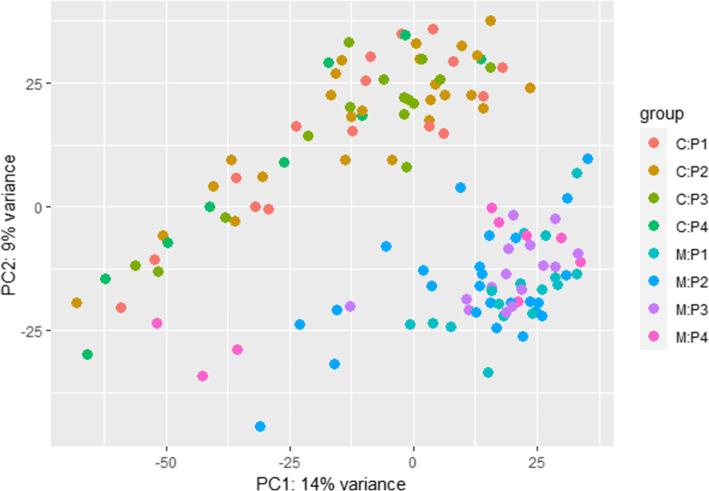
PCA plot of colostrum (C) and mature milk (M) transcripts from dams in parities 1–4

The principal component analysis (PCA) plot (Fig. 9) showed that colostrum and mature milk transcript expression profiles seem to fall into distinct clusters, while there was no clear clustering of samples by parity. After multiple testing correction, we identified 169 differentially expressed transcripts (DET) for the milk type x parity interaction, 4783 DET for the milk type main effect, and 9639 DET for the parity main effect (Tables S6, S7 and S8). Table 4 shows the classifications of DET. The DET set for the milk type main effect was comprised of 2479 known transcripts, 2132 novel coding transcripts, and 172 novel lncRNA, while the interaction DET set included 85 known transcripts and 80 and 4 novel coding transcripts and lncRNA, respectively. The 25 most significant DET for milk type and interaction are given in Tables 5 and 6, respectively. *P*-values of transcripts were aggregated for each gene loci to obtain DEG. A total of 1667 DEG were identified for the milk type main effect, and 33 DEG were identified for the milk type x parity interaction (Tables S9 and S10).

**Table 4:** Classifications of DET for milk type main effect and milk type x parity interaction

	Milk Type	Milk Type x Parity
**Known Transcripts**	2479	85
**Novel Coding Transcripts**	2132	80
**Novel lncRNA**	172	4
**Total**	4783	169

**Table 5:** Twenty-five most significant DET associated with milk type. Significance was ranked using FDR-adjusted *P*-value

Transcript	Gene	Gene Description
rna45537^a^	CCDC71L	Coiled-coil domain containing 71 like
rna5080^a^	ALDH1A1	Aldehyde dehydrogenase 1 family member A1
rna54008^b^	KCNH8	Potassium voltage-gated channel subfamily H member 8
rna19955^a^	C4H8orf46	Vexin (VXN)
rna70598^b^	LOC100513767	Colostrum trypsin inhibitor-like
rna37492^a^	GZMB	Granzyme B
rna42732^a^	THRSP	Thyroid hormone responsive
rna8225^b^	LRP4	LDL receptor related protein 4
MSTRG.255032.1^a^	LOC110256328	Uncharacterized ncRNA
rna57086^a^	BCHE	Butyrylcholinesterase
rna4906^a^	SETDB2	SET domain bifurcated histone lysine methyltransferase 2
MSTRG.313167.1^b^	MSTRG.313167	Novel gene loci
rna48308^b^	CALML5	Calmodulin like 5
MSTRG.211924.1^b^	KIF26B	Kinesin family member 26B
rna56663^a^	LOC100739719	Tetraspanin-6
rna16073^b^	SOWAHC	Sosondowah ankyrin repeat domain family member C
rna75068^b^	COL4A5	Collagen type IV alpha 5 chain
rna8510^b^	ABTB2	Ankyrin repeat and BTB domain containing 2
rna5531^a^	STAB1	Stabilin 1
rna2697^a^	VPS33B	VPS33B late endosome and lysosome associated
rna29012^b^	TRAPPC6A	Trafficking protein particle complex subunit 6A
rna44370^b^	FMO2	Flavin containing dimethylaniline monooxygenase 2
rna44593^a^	PIGR	Polymeric immunoglobulin receptor
rna8754^a^	SAA3	Serum amyloid A-3 protein
rna37493^a^	GZMH	Granzyme H

^a^ Indicates up-regulation in mature milk

^b^ Indicates down-regulation in mature milk

**Table 6:** Twenty-five most significant DET associated with milk type x parity interaction. Significance was ranked using FDR-adjusted *P*-value

Transcript	Gene	Gene Description
rna22238	AHCYL1	Adenosylhomocysteinase like 1
rna27866	LOC100621677	Dipeptidase 3 (DPEP3)
MSTRG.208683.14	GRB10	Growth factor receptor bound protein 10
rna14076	POR	P450 (cytochrome) oxidoreductase
rna62377	ANXA7	Annexin A7
rna69234	PCM1	Pericentriolar material 1
MSTRG.337836.10	EXOC4	Exocyst complex component 4
rna62750	ADIRF	Adipogenesis regulatory factor
rna69254	PCM1	Pericentriolar material 1
rna16978	EHBP1	EH domain binding protein 1
MSTRG.114444.62	LMNTD1	Lamin tail domain containing 1
rna31913	PTP4A2	Protein tyrosine phosphatase 4A2
MSTRG.329474.6	RRBP1	Ribosomal binding protein 1
MSTRG.86648.4	PUF60	Poly(U) binding splicing factor 60
rna56801	WWTR1	WW domain containing transcription regulator 1
MSTRG.253426.1	SELENOT	Selenoprotein T
MSTRG.40523.3	PDCL	Phosducin like
rna50113	GPS1	G protein pathway suppressor 1
MSTRG.341587.6	SCRN1	Secernin 1
rna77208	EIF5	Eukaryotic translation initiation factor 5
MSTRG.290003.24	GPAM	Glycerol-3-phosphate acyltransferase, mitochondrial
MSTRG.266678.8	NRIP1	Nuclear receptor interacting protein 1
MSTRG.118470.4	TXNRD1	Thioredoxin reductase 1
MSTRG.373117.37	LOC102158401	Collagen alpha-1(l) chain-like
MSTRG.221153.6	STARD13	StAR related lipid transfer domain containing 13

### Gene ontology and pathway analysis

Gene ontology (GO) analysis of the DEG indicated that genes associated with the milk type main effect were predominantly involved in binding (37.5%), catalytic activity (30.5%), molecular function regulation (15.8%), and transporter activity (8.2%). A total of 250 biological process, 25 molecular function, and 54 cellular component GO terms were significantly enriched in this gene set (Table S11). Additionally, 3 KEGG pathways were significantly enriched.

Like the milk type main effect genes, DEG for the milk type x parity interaction were involved in binding (45.5%), catalytic activity (27.3%), molecular adapter activity (9.1%), molecular function regulation (9.1%), and transporter activity (9.1%). No GO terms or pathways were significantly enriched in this DEG set.

## Discussion

Milk production, milk composition, milk intake, and milk digestibility are all major limiting factors in the growth and survival of a sow’s litter. Knowledge of porcine milk composition, as well as understanding genetic factors underlying its variation, is a matter of ongoing interest. In this study, we performed the first exhaustive characterization of the porcine milk transcriptome derived from whole milk samples. The goal was to characterize and compare transcriptomic profiles of samples collected during early and mid-lactation from dams across different parities. This study was the first in a series of studies aimed at exploring the molecular profile of the piglet’s neonatal diet.

Total RNA was isolated from 130 fresh whole milk samples (65 colostrum and 65 mature milk) from dams across four parities. In most milk transcriptome studies, milk is fractionated, and RNA is extracted from somatic cells, milk fat, or whey. Total RNA concentrations tend to be higher in the milk fat and somatic cells than in the whey fraction, while RNA integrity of somatic cells is higher than those of milk fat and whey [33, 34]. Low RIN values in this study (average RIN = 4.0) are likely due to the presence of small amounts of cytoplasmic material in milk fat globules [35], bacteria and small RNA (miRNA) in the fat fraction [36], and degraded and/or free RNA. Each milk fraction has its own place in research settings. The advantages and disadvantages of each RNA source has previously been summarized [32]. In this study, we chose to utilize whole milk samples in order to capture the broader transcriptomic signatures of porcine colostrum and milk. We were able to process the samples much more quickly than had we fractionated the milk, and our sample represents the entirety of what is being ingested by the growing piglet.

Libraries were sequenced to an average depth of 46 million reads per library. A depth of 40 million reads is considered sufficient for reliable detection of major splice isoforms for abundant and moderately abundant transcripts [37]. When generating our sequence data, we targeted a depth of 50 million reads per library. However, there was considerable variation in sequence depth across libraries. Some of this variation can be attributed to technical aspects of next-generation sequencing (NGS) technology, such as the stochasticity of sequencing, RNA quality, and library preparation.

A total of 70,841 transcripts were identified in this study, of which approximately 60% are annotated in the current swine genome build. Transcripts corresponded to 17,910 unique gene loci, including 17,296 known porcine genes. The number of expressed genes is comparable to those reported in similar studies in sheep [25] and goat [24]. A smaller number of expressed genes (~ 13,500) was reported in the buffalo milk transcriptome [26]. This discrepancy is likely to due to the swine, sheep, and goat reference genomes being more complete and of higher quality.

As expected, cells in our whole milk samples appeared to be a heterogeneous population of immune, epithelial, stromal, and stem cells (Table 3; Fig. 8). Epithelial cells represented the largest subset of the cell population in all samples, on average 85% of the cell population per sample. This is consistent with findings in bovine milk [31]. Immune cells were the second most abundant cell type, comprising an average of 14 and 9% the colostrum and mature milk cell populations, respectively. In general, stromal cells were more highly expressed in colostrum. In particular, adipocytes (characterized by the FABP4 marker) accounted for nearly 2% of colostrum cell populations. Adipocytes release the hormone leptin in the presence of insulin, which is present in colostrum and mature milk. Previous studies have shown a decrease in leptin concentration in milk across lactation stages in swine [38], human [39], and cattle [40]. Hemopoietic stem cells accounted for approximately 1% of the cell population in both colostrum and mature milk, differing from findings in human where hemopoietic stem cells were significantly higher in mature milk compared to colostrum [41].

Previous milk transcriptome studies in livestock have used sequencing of milk somatic cells as a proxy for the mammary gland to study the lactation process. Recent studies have indicated that RNA originating from multiple cell types present in milk can withstand harsh environments, such as the digestive system, and transmit regulatory molecules from maternal to neonate [15–17]. Hence, transcriptome profiling of whole milk samples, which is reflective of the combined cell populations, is needed to understand these mechanisms. Most of the stable, bioactive RNA in milk reported in the literature has been miRNA [17]. However, stable mRNA, alpha S2-casein (CSN1S2), beta-casein (CSN2), and beta-lactoglobin (BLG), have been reported in cattle [16]. These three mRNA were also found to be expressed in both colostrum and mature milk samples in this study. Additional studies are needed to confirm whether these mRNA can function in the piglet gastrointestinal tract.

Among the top expressed genes were *CSN3, CSN2, CSN1S1, LALBA, FASN, EEF1A1, PAEP, TPT1, FABP3, XDH, PIGR,* and *SAA3* (Table 2; Fig. 7)*,* which have been previously identified among the top expressed genes in milk samples from other species [10, 24–26, 42]. As expected, many of the top expressed genes were related to biosynthesis of milk proteins. Expression levels of *CSN2, CSN3, CSN1S1, LALBA,* and *PAEP,* which encode for the synthesis of the main milk proteins casein and whey, increased from early to mid-lactation stages. A similar gene expression pattern has been identified in a previous swine study [43], as well as in goat [24], cattle [42], and sheep [25]. High expression of the *EEF1A1* gene is also related to high levels of milk protein synthesis, as *EEF1A1* is one of the most abundant protein synthesis factors [24]. Consistent with results in buffalo [26], ribosomal protein *RPLP0* was among the top expressed genes in colostrum and exhibited a slight decrease in expression during mid-lactation.

In addition to milk protein synthesis genes, genes associated with milk fat were among the top expressed genes, and their expression increased from early to mid-lactation. Milk fat composition is known to influence piglet growth and development [44]. The *FABP3* gene, which is involved in the uptake and transport of fatty acids, has been linked to milk fat synthesis in cattle [45]. *FASN* is directly involved in most of the short and medium-chain fatty acids in milk [46], and *PLIN2* is involved in the formation of the lipid droplet in milk [47].

DET were determined for the milk type by parity interaction, as well as both the milk type and parity main effects. DET for the parity main effect are presented for completeness (Table S8), but the discussion will be restricted to DET/DEG for the milk type main effect and milk by parity interaction, as the objective of this study was to investigate transcriptomic differences between colostrum and milk.

Several of the most significant DET were associated with genes involved in milk fat synthesis and immunity (Tables 4 and 5). Transcripts *rna42732* (*THRSP* gene) and *rna62377* (*ANXA7* gene) are milk fat synthesis genes among the most significant DET. *THRSP*, thyroid hormone responsive, is a crucial protein for cellular de novo lipogenesis and has been shown to play an important role in lipogenesis in the mammary epithelial cell [48, 49]. Expression of milk fat synthesis transcripts was up-regulated in mature milk samples compared to colostrum, which agreed with expression patterns observed across bovine lactation stages [50]. Our results are consistent with the finding that the transition from swine colostrum to mature milk is marked by a shift from high protein contents to high fat and lactose contents [51].

Transcript *rna70598,* associated with the colostrum trypsin inhibitor-like gene (*LOC100513767*), was found to be significantly differentially expressed for the milk type main effect. Moreover, its expression was over 7-fold higher in colostrum than mature milk. Trypsin secreted by the small intestine can degrade colostral antibodies, and swine immunoglobulins, such as IgG and IgA are susceptible to trypsin degradation [52]. Colostral trypsin inhibitor helps protect these immunoglobulins without preventing the digestion of other milk proteins. In addition, DET associated with granzymes *GZMB* (transcript *rna37492*) and *GZMH* (transcript *rna37493*) were among the most significant DET. Granzymes are serine proteases and six of the twelve members of the granzyme family (A, B, H, K, and M) have been identified in the swine genome [53]. Presence of these proteins in milk leukocytes would indicate the existence of activated or memory t-cells which are likely actively fighting pathogenic cells which may be of importance for either the infant or the protection of the mammary gland [54].

In this study, DEG were identified by aggregating *P*-values across transcripts associated with each gene via the Lancaster method, rather than using gene read counts directly. Using this approach not only maintains both transcript and gene-level resolution, but also bypasses issues of different variances and directions of change across constituent transcripts. This method outperforms other gene-level methods and provides a coherent analysis between transcripts and genes [55].

One of the major aims of this study was to evaluate DEG between lactation stages across different parities. Progeny born to multiparous sows generally exhibit superior growth performance compared to those born to primiparous sows. However, colostrum and milk composition profiles (immunoglobulin, protein, fat, lactose, and net energy) are highly similar across parities [6]. Results from this study support this finding, as very few gene expression differences were identified in the milk type by parity interaction. Only 33 DEG were identified for the milk type by parity interaction and only a clear separation in milk type was exhibited in the PCA analysis (Fig. 9).

Glucose transport is a major precursor to lactose synthesis, which is synthesized in the Golgi vesicle of mammary secretory alveolar epithelial cells during lactation [56]. Glucose-6-phosphate transporter *SLC37A2* and glucose transporter *SLC2A5* were identified as DEG for the milk type main effect. Glucose transport across the plasma membrane of mammalian cells is carried out by two distinct processes one of which involves glucose transporters from the *GLUT* gene family (encoded by *SLC2A* genes) and the other which involves glucose transporters from the *SGLT* family (encoded by *SLC5A* genes). Both the *SLC2A5* and *SLC37A2* genes were up-regulated in colostrum. Crisá et al. [24] identified significant up-regulation of members of the *SLC2A* gene family and polysaccharide and glycosamino-glycan binding molecular function to be enriched in goat colostrum samples compared to mature milk.

Members of the *SLC35* gene family encode nucleotide sugar transporters localizing at the Golgi apparatus and/or the endoplasmic reticulum. These transporters transport nucleotide sugars pooled in the cytosol into the lumen of these organelles, where most glycoconjugate synthesis occurs [57]. Currently, the *SLC35* gene family is comprised of 31 genes which are divided into 7 subfamilies, *SLC35A* to *SLC35G* [58]. GDP-fucose transporters *SLC35C1* and *SLC35C2* were identified as DEG for the milk type main effect, with *SLC35C2* up-regulated in colostrum and *SLC35C1* down-regulated. Several other members of the *SLC35* family, *SLC35B2, SLC35D1, SLC35E1, SLC35E3,* and *SLC35G1,* were statistically significant but were filtered out based on our log-fold change criteria. Crisà et al. [24] identified 3 DEG from the *SLC35* family that were up-regulated in goat colostrum compared to mature milk, as well as enrichment of glycosaminoglycan binding molecular in colostrum. Consistent with this result, we also identified the enrichment of glycosaminoglycan binding molecular function, with 22 of the 118 annotated genes associated with the GO term being present in our milk type DEG set.

The JAK-STAT pathway regulates lactation [59]. The main gene families in the pathway are Janus kinases (*JAK*) and the signal transducers and activators of transcription proteins (*STAT*). Members of the *JAK* family, *JAK1, JAK2, JAK3,* and *TYK2*, have been linked to cytoplasmic domains of diverse cytokine receptors [60], while members of the *STAT* family, *STAT1–4*, *STAT5A*, *STAT5B*, and *STAT6*, are involved in cell growth, differentiation, apoptosis, and mammary gland development. Members of both families have been associated with bovine milk production [61]. *JAK3* was significantly differentially expressed for the milk type main effect, with increased expression in colostrum, while *JAK2* was statistically significant but was filtered out of our DEG list due to thresholding on log-fold change (log2 fold change = 1.3). A single nucleotide polymorphism (SNP) in the *JAK2* gene has previously been associated with milk, protein, and fat yields in bovine milk production [62].

**Fig. 10:**
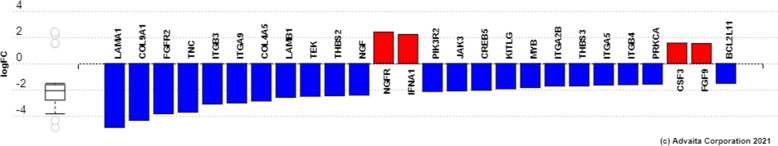
Log2 fold change (mature milk vs. colostrum) of milk type main effect DEG in the PI3K-AKT signaling pathway. Image was produced by the iPathwayGuide software (Advaita Bio, http://advaitabio.com/ipathwayguide)

Twenty-six genes from the PI3K-Akt pathway, which lies within the JAK-STAT pathway, were found to be differentially expressed. The PI3K-Akt pathway is important for the synthesis of lactose and lipids, as well as glucose transport [63]. Although not statistically significant after FDR-correction, this pathway had a nominal *P*-value of 0.029 in the enrichment analysis. The PI3K-Akt pathway is a key signaling node for lactogenic expansion and differentiation of the luminal mammary epithelium, as numerous signaling pathways that regulate lactogenic development converge on PI3K-Akt, including the insulin-like growth factor 1 receptor (*IGF1R*), *RANKL* and *RANK*, integrins, and *PRLR*-to-*JAK2*-to-*STAT5A* pathways [64]. In general, expression of DEG in the PI3K-Akt pathway was up-regulated in colostrum (Fig. 10). The PI3K-Akt pathway was also identified as major pathway enriched in human and bovine colostrum [65].

Several GO terms related to immune response, particularly leukocyte differentiation, leukocyte migration, regulation of immune system process, and humoral immune system process, were significantly enriched in the DEG for the milk type main effect (Tables S11 and S12). Nearly all of the DEG associated with these GO terms were up-regulated in colostrum. This finding is consistent with the immunoglobulins and immune cells being transferred to the neonate via colostrum [66]. In pigs, the epitheliochorial nature of the placenta prohibits transfer of maternal immune cells and immunoglobulins to the fetus, and thus, the piglet relies on the successful absorption of colostral components to acquire maternal immunity [67]. Proinflammatory cytokines play an important role in the development of the neonatal immune system by mediating the early local and systemic responses to microbial challenges [68]. A total of 16 DEG were associated with cytokine secretion, including interleukin 21 receptor (*IL21R*), interleukin 27 receptor A (*IL27RA*), and tumor necrosis receptor superfamily members 1B (*TNFRSF1B*). Several other genes in these gene families were shown to be up-regulated in early porcine lactation by Palombo et al. [20]. This was consistent was our findings as all cytokine secretion genes except *CD36*, *CIDEA, F2RL1, TLR6*, and *BTN1A1* were up-regulated in colostrum (Table S12).

**Fig. 11:**
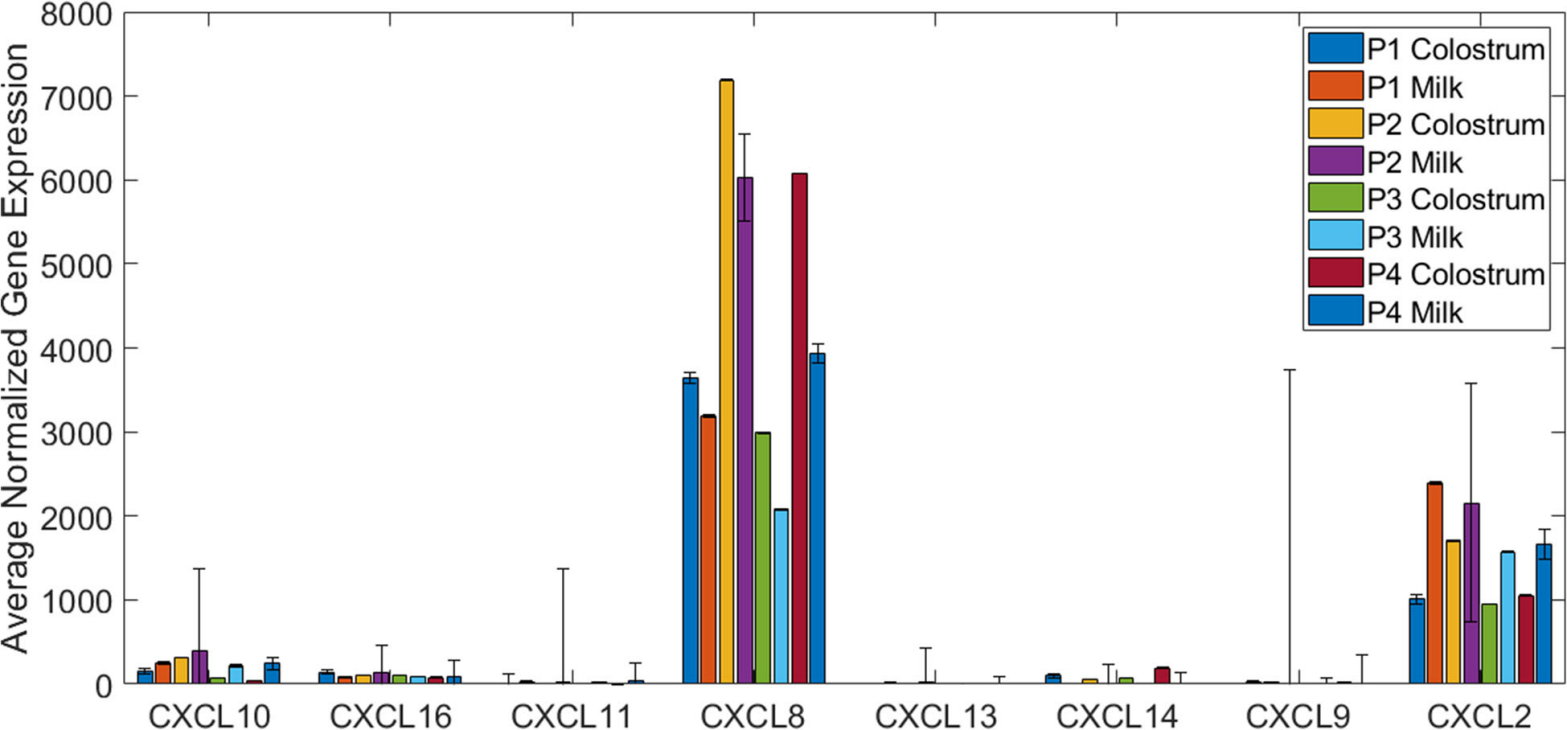
Average gene expression values of genes in chemokine superfamily (*CXC*)

Antimicrobial proteins naturally present in colostrum and milk can kill and inhibit a broad spectrum of bacteria [69]. Milk is also known to exert chemotactic activity on neutrophils [70], an important innate host defense against microorganisms. The chemokine superfamily encodes secreted proteins involved in immunoregulatory and inflammatory processes. The CXC chemokine ligand 14 (*CXCL14*), which encodes a chemokine antimicrobial protein [71], was up-regulated in colostrum samples (Fig. 11). Many of the other main chemokines (*CXCL2, CXCL8, CXCL9, CXCL10, CXCL11, CXCL13, CXC14*, and *CXCL16*) were expressed in our samples. Interleukin 8/CXC ligand (*CXCL8*) was the most highly expressed chemokine across our samples. It has also been reported to be highly expressed in human milk [72–74]. In human milk, the expression of *CXCL8* was highest in the immediate postpartum period and decreased over the first week of lactation [74]. A similar pattern was observed in our samples, with markedly higher expression of *CXCL*8 in colostrum compared to mature milk across all parities. The second highest expressed chemokine in our samples was growth-related oncoprotein beta (*CXCL2*). In parity 1 and parity 3 samples, expression of *CXCL2* was higher in mature milk samples (*P* = 0.0023 for P1 and *P* = 0.0160 for P3; paired T-test). Though not statistically significant, the average expression values for CXCL2 were also higher in mature milk in parities 2 and 4. Although expression of *CXCL2* was found to be high in human milk samples, it did not change with time postpartum [74]. In bovine milk samples, it was reported that compared to other chemokines, concentrations of *CXCL2* are generally low and decrease sharply after the onset of lactation [70]. Palombo et al. [20] identified significant up-regulation of *CXCL2* and *CXCL10* in day 1 postpartum swine mammary gland samples compared to mammary samples taken before parturition. We found that both *CXCL2* and *CXCL10* increased in expression with time postpartum. Our results differed from the results in [20] in that *CXCL8* was the most abundantly expressed chemokine in our colostrum and mature milk samples, and *CXCL3* was not expressed. One factor contributing to this discrepancy was the use of the improved reference genome (Sscrofa 11.1), where many of the gaps and misassemblies present in the Sscrofa 10.2 genome build were resolved and the annotation was significantly improved. These results suggest that chemokine ligands may play an important role in the transition from colostrum to mature milk in swine, likely helping prompt recruitment of neutrophils.

## Conclusions

Porcine milk and colostrum are complex biofluids that nourish the neonate and protect it from pathogens and disease. Recent research has shown that in addition to the major nutrient components, such as carbohydrates, lipids, and proteins, other bioactive components, including but not limited to exosomes, oligosaccharides, and bacteria, are present in porcine milk. Understanding both the nutritional and non-nutritional components of porcine milk is essential for improved pig production. Some vital questions that need to be addressed are: 1) What is the sow’s genetic contribution to milk composition? 2) What bacteria are present in the mammary gland, milk, and piglet gut, and what is the source of these bacteria? 3) Do different milk oligosaccharide profiles contribute to the microbiome and immunity in the piglet GI tract? This study is a subset of a larger study aimed at addressing these questions. Our findings have produced several highly specialized and functional candidate genes that may contribute to postnatal development and growth of piglets, as well as lactation in the sow. A deeper understanding of these genes could provide a coherent approach to genetically regulate milk composition in the future.

## Methods

### Population and sampling

A four-breed composite line (Maternal Landrace × High-lean Landrace × Duroc × Yorkshire) maintained at the U.S. Meat Animal Research Center (USMARC) for at least 18 generations was used for the collection of data in this project and has been previously described [75, 76]. Litter sizes were adjusted within 48 h of farrowing to ensure litters were approximately equal in size but did not exceed the number of functional teats. Mammary excretion samples were collected on day of farrowing (d 0; colostrum) and again on day 10 post-farrowing (d 10; mature milk) from a total of 65 dams, 16 first parity (P1), 25 s parity (P2), 15 third parity (P3), and 9 fourth parity (P4). The power calculation for this experiment was conducted using the online RNASeqSampleSize tool ([77]; http://cqs.mc.vanderbilt.edu/shiny/RnaSeqSampleSize/). The power of using 65 animals to detect ~ 1000 DEG, with maximum dispersion 0.5 and minimum fold change of 2.82, at FDR level 0.05 from 17,740 expressed genes was found to be 0.99. After sample collection, animals remained at USMARC and progressed through the breeding system according to standard operating procedures.

In most cases, no external stimulant (i.e., oxytocin) was needed to collect colostrum at time of farrowing, as farrowing stimulates endogenous oxytocin production and milk letdown activity. However, if enough colostrum could not be collected within 10–30 min, an intramuscular injection of oxytocin (20 IU) was administered to stimulate colostrum letdown. Teats were sprayed with iodine (5%) and ethanol (70%) and wiped clean with a chem-wipe, and then 10 mL of colostrum was collected manually from the third and fourth teat on one side of the sow. On d 10, piglets were separated from the sow for approximately 1 h, and sows were given an intramuscular injection of 20 IU oxytocin to stimulate milk letdown. Teats were cleaned, and 10 mL of milk was collected manually from the third and fourth teat on one side of the sow. Fresh samples were transported to the laboratory on ice. Samples (250 μL) were aliquoted into individual lysis D matrix tubes (MP Biomedicals, LLC, Solon, OH) with 1 mL TRIzol reagent (Invitrogen, Thermo Fisher Scientific, Waltham, MA) and stored at − 80 °C until RNA isolation.

### RNA isolation and sequencing

RNA was isolated using the FastPrep-24 5G Instrument (MP Biomedicals, LLC) with cryogenic lysis. Briefly, RNA was isolated by high-speed cellular disruption using multi-directional, simultaneous bead beating of sample material (i.e., colostrum or milk) with a cool adapter for cryogenic lysis at 6.0 m/sec for 40 s. Lysed samples were transferred into a clean tube, and completion of isolation occurred following manufacturer’s recommended protocol for TRIzol. The final RNA pellet was dried at RT for 10 min and resuspended in 30 μL water (Invitrogen UltraPure DNase/RNase-free, Thermo Fisher Scientific). RNA was quantified using a NanoDrop UV-Vis spectrophotometer (Thermo Fisher Scientific) and RNA integrity was assessed using an Agilent Bioanalyzer System (Agilent, Santa Clara, CA).

Total RNA samples extracted from colostrum or milk were prepared for RNA sequencing with the TruSeq Stranded Total RNA with Ribo-Zero Gold sample preparation kit (Illumina, San Diego, CA) following the guidelines of the manufacturer. Libraries were quantified with RT-qPCR using the NEBNext Library Quant Kit (New England Biolabs, Inc., Beverly, MA, USA) on a CFX384 thermal cycler (Bio-Rad, Hercules, CA, USA), and the size and quality of the library was evaluated with an Agilent Bioanalyzer DNA 1000 kit (Santa Clara, CA, USA). The libraries were diluted to 4 nM with Illumina RSB. Libraries were paired-end sequenced with 150 cycle high output sequencing kits on an Illumina NextSeq 500 instrument.

### Processing RNA-Seq data

Alignment of RNA-Seq reads was carried out as follows. First, quality of the raw paired-end sequence reads in individual fastq files was assessed using FastQC (Version 0.11.5; www.bioinformatics.babraham.ac.uk/projects/fastqc), and reads were trimmed to remove adapter sequences and low-quality bases using the Trimmomatic software (Version 0.35) [78]. The remaining reads were mapped to the Sscrofa 11.1 genome assembly (NCBI accession AEMK00000000.2) using Hisat2 (Version 2.1.0) [79] with default parameters.

Mapped transcripts were assembled for each library using Stringtie (Version 1.3.3) [80]. The NCBI Sscrofa 11.1 reference annotation (Release 106) was used to guide the assembly process. Transcripts from all samples were merged using Stringtie merge mode to build a consensus set of transcripts.

### Identification and characterization of novel transcripts

Transcript expression levels were quantified for each library using fragments per kilobase of exon per million mapped reads (FPKM) [81]. Transcripts expressed in a single sample, and transcripts with FPKM < 0.3 in all samples were removed. Gffcompare (Version 0.11.2) [82] was used to compare the list of assembled transcripts with the *S. scrofa* reference annotation (NCBI Release 106). Transcripts overlapping known transcript classes in the reference annotation (gffcompare class code ‘=’) were assigned to the appropriate annotation class, while transcripts with gffcompare class codes ‘x’ (exonic overlap on the opposite strand), ‘i’ (fully contained in reference intron), ‘j’ (multi-exon with at least one junction match), and ‘u’ (unknown, intergenic) were considered to be potential novel transcripts.

A modified version of the discovery pipeline described in Cai et al. [83] was used to further filter transcripts and classify novel transcripts (Fig. 2): (i) Filter out transcripts with short lengths (< 200 bp) and single exons; (ii) ORF obtained using TransDecoder (Version 5.5.0; https://github.com/TransDecoder/TransDecoder/wiki). Transcripts with no predicted ORF were filtered out, and transcripts ORF length ≥ 120 amino acids were considered protein-coding. (iii) Protein coding potential assessed using CPC2 (Version 2.0) [84], PLEK (Version 1.2) [85], and CNIT [86]; (iv) Transcripts were translated to amino acid sequences using the Transeq utility from EMBOSS (https://www.ebi.ac.uk/Tools/st/emboss_transeq/), and HMMER [87] was used to search for known protein domains against the Pfam database (Release 33.1) [88]. Transcripts with significant Pfam hits (E-value < 10.0) were classified as protein-coding. After steps (iii) and (iv), transcripts with no significant Pfam hits, CPC2 classification “coding”, PLEK score > 0, and CNIT > 0 were classified as protein-coding, and transcripts with no significant Pfam hits, CPC2 classification “noncoding”, PLEK score < 0, and CNIT score < 0 were classified as non-coding. All other transcripts were discarded, as their coding potential was ambiguous.

The BLASTN algorithm from the BLAST+ package [89] was used to identify homology between (1) novel lncRNA and the NONCODE database (Version 5) and (2) novel mRNA and the NCBI Non-redundant Nucleotide database (nt; Version 5, https://ftp.ncbi.nlm.nih.gov/blast/db/). BLASTN was run with default parameters, and an E-value cutoff of 10.0 was used to define homologous sequences.

### Differential expression and functional analyses

Raw read counts for the 70,841 transcripts and the 17,910 corresponding genes were normalized using DESeq2 (Version 1.26) [90]. Gene expression distributions were computed for colostrum and mature milk samples by averaging the normalized expression values across samples. The PCA plot, using variance stabilizing transform of normalized read counts, was generated using the plotPCA function from the DESeq2 package. Differential expression analysis of transcripts was performed using DESeq2 with the following generalized linear model:

$$ Y= Type+ Parity+ Type\ x\  Parity. $$

Transcripts with FDR-adjusted *P*-value ≤0.01 were considered DET for the type x parity interaction. Transcripts that were not DET for the interaction term with FDR-adjusted *P*-value ≤0.01 were considered DET for the parity main effect. Transcripts that were not DET for the interaction term with |log2 fold change| ≥ 2 and FDR-adjusted *P*-value ≤0.01 were considered DET for the milk type main effect.

Transcript *P*-values were aggregated for each gene using the Lancaster method [91] to generate gene-level analysis. This approach has been described in detail by Yi et al. [55]. Briefly, the Lancaster method is an extension of the Fisher method [92] for *P*-value aggregation, where under the null hypothesis that all genes have zero effect, the test statistic $T={\sum}_{i=1}^K{\phi}_{w_i}^{-1}\left({p}_i\right)$ follows a chi-squared distribution with $df={\sum}_{i=1}^K{w}_i$. Here, *K* denotes the number of transcripts associated with the gene, *w*_1_, …, *w*_*K*_ a set of weights for the transcript *P*-values *p*_1_, …*p*_*K*_, and ${\phi}_{w_i}^{-1}$ the inverse CDF of the gamma distribution with shape parameter ${\alpha}_i=\frac{w_i}{2}$ and scale parameter *β* = 2. Here, the baseMean parameter from the DESeq2 output was used as *w*_*i*_ in the Lancaster method. Aggregated *P*-values were corrected using the Benjamini-Hochberg method. Genes with FDR-adjusted *P*-value ≤0.01 were considered DEG. DEG for the milk type x parity interaction were removed from the milk type main effect DEG set. Log2-fold changes (log2FC; mature milk vs. colostrum) were computed for each of the genes using DESeq2, and genes with |log2FC| ≤ 1.5 were filtered out of the milk type main effect DEG set.

Enrichment analysis of gene function and cellular pathways was performed for DEG using the iPathwayGuide software (Version 2012; Advaita Bio, http://advaitabio.com/ipathwayguide) with the default *M. musculus* data as background. For GO analysis, an over-representation test, based on a hypergeometric distribution, was used to compute the statistical significance of observing more than the expected number of DEG. A GO term was considered statistically significant at FDR-corrected *P* ≤ 0.05. Pathway over-representation analysis was performed by comparing the number of affected genes associated with a pathway between groups. Pathways were considered statistically significant at FDR-corrected *P* ≤ 0.05.

## Supplementary Information

**Additional file 1: Table S1.** Sequencing statistics. Number of raw sequence reads, number of reads mapping to the *S. scrofa* 11.1 genome assembly, and percentage of mapped reads for each of the sequenced libraries.

**Additional file 2: Table S2.** Summary of reads mapping to RNA regions of the swine genome. Number of raw sequence reads mapping to annotated RNA regions in the *S. scrofa* 11.1 genome assembly. RNA classifications are based on the NCBI *S. scrofa* annotation (Release 106).

**Additional file 3: Table S3.** Novel lncRNA annotation. Annotation is given in GTF format. LincRNA denotes intergenic lncRNA, ilncRNA denotes intronic RNA, lncNAT denotes natural antisense lncRNA, and isolncRNA denotes novel isoform lncRNA.

**Additional file 4: Table S4.** Novel mRNA annotation. Annotation is given in GTF format.

**Additional file 5: Table S5.** BLAST search results for novel lncRNA against NONCODE databases. BLAST hits for novel lncRNA searched against NONCODE databases for multiple species. Entries in the species column indicate the top BLAST hit for the transcript in the given species.

**Additional file 6: Table S6.** Analysis of differentially expressed transcripts (DET) for the milk type x parity interaction. DET are highlighted in blue.

**Additional file 7: Table S7.** Analysis of differentially expressed transcripts (DET) for the milk type main effect. DET are highlighted in blue. Transcripts colored gray were filtered out for being significant for the milk type x parity interaction or for having |log2 fold change| < 2. Log2 fold change is for milk vs. colostrum comparison.

**Additional file 8: Table S8.** Analysis of differentially expressed transcripts (DET) for the parity main effect. DET are highlighted in blue. Transcripts colored gray were filtered out for being significant for the milk type x parity interaction.

**Additional file 9: Table S9.** Analysis of differentially expressed genes (DEG) for the milk type x parity interaction. DEG are highlighted in blue.

**Additional file 10: Table S10.** Analysis of differentially expressed genes (DEG) for the milk type main effect. DEG are highlighted in blue. Genes colored gray were filtered out for being significant for the milk type x parity interaction or for having |log2 fold change| < 2. Log2 fold change is for milk vs. colostrum comparison.

**Additional file 11: Table S11.** Gene ontology (GO) term and pathway analysis for the milk type main effect. Biological process (BP), molecular function (MF), cellular component (CC), and KEGG pathway results are shown in separate tabs.

**Additional file 12: Table S12.** Milk type DEG associated with various immune response GO terms. GO terms are given in separate tabs. DEG associated with the GO term and their log2 fold changes (milk vs colostrum) are shown.

**Additional file 13: Figure S1.** Number of transcripts falling into each Gffcompare class code based on the NCBI *S. scrofa* 11.1 reference annotation. For class code definitions see https://ccb.jhu.edu/software/stringtie/gffcompare.shtml

## Data Availability

Sequence data used in this study was submitted to the National Center for Biotechnology Information Sequence Read Archive (NCBI SRA) with Accession Number PRJNA640341.

## Abbreviations

IgG: Immunoglobin
RNA: Ribonucleic acid
NGS: Next-generation sequencing
MEC: Milk epithelial cells
lncRNA: Long non-coding RNA
bp: Base pairs
RNA-Seq: RNA-sequencing
DEG: Differentially expressed gene
mRNA: Messenger RNA
miRNA: Micro RNA
miscRNA: Miscellaneous small non-coding RNA
circRNA: Circular RNA
ncRNA: Non-coding RNA
ORF: Open reading frame
lincRNA: Long intergenic non-coding RNA
ilncRNA: Identified unknown long non-coding RNA
lncNAT: Long non-coding natural antisense transcripts
isolncRNA: Novel isoform non-coding RNA
SNP: Single nucleotide polymorphism
PCA: Principal component analysis
DET: Differentially expressed transcript
GO: Gene ontology
USMARC: U.S. Meat Animal Research Center
NCBI: National Center for Biotechnology Information
SRA: Sequence Read Archive
FPKM: Fragment per kilobase of exon per million mapped reads
