# Supplementary material for: Characterization and comparative analysis of transcriptional profiles of porcine colostrum and mature milk at different parities
Source: BMC Genom Data. 2021 Aug 10;22:25. doi: 10.1186/s12863-021-00980-5 (PMC8353812; doi:10.1186/s12863-021-00980-5)

**Figure S1.** Number of transcripts falling into each Gffcompare class code based on the NCBI Sus scrofa 11.1 reference annotation. For class code definitions see https://ccb.jhu.edu/software/stringtie/gffcompare.shtml


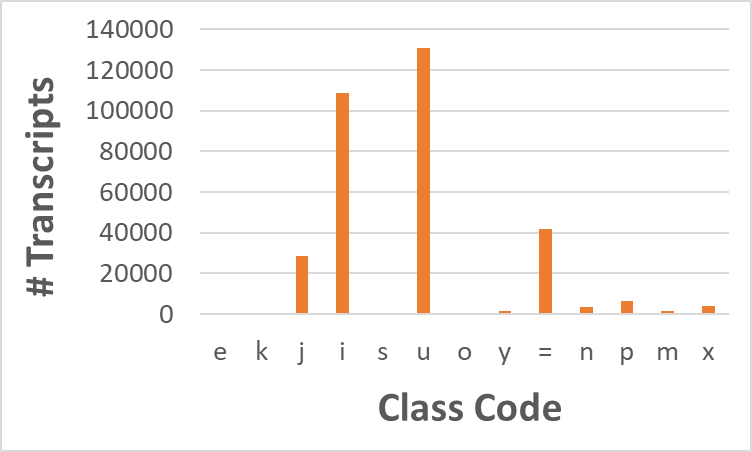

Supplement: Supplementary file 13 — Additional file 13: Figure S1. Number of transcripts falling into each Gffcompare class code based on the NCBI S. scrofa 11.1 reference annotation. For class code definitions see https://ccb.jhu.edu/software/stringtie/gffcompare.shtml [file 12863_2021_980_MOESM13_ESM.docx]
